# The Role of Phenotypic Plasticity and Within‐Environment Trait Variability in the Assembly of the Nectar Microbiome and Plant–Microbe–Animal Interactions

**DOI:** 10.1002/ece3.71059

**Published:** 2025-03-02

**Authors:** Sergio Quevedo‐Caraballo, Sergio Álvarez‐Pérez

**Affiliations:** ^1^ Department of Animal Health Complutense University of Madrid Madrid Spain

**Keywords:** bacterium, community assembly, floral nectar, phenotypic plasticity, plant–microbe–animal interaction, yeast

## Abstract

The study of the rules that govern the relationship between phenotypic plasticity, genetic structure, and ecological success has traditionally focused on animals, plants, and a few model microbial species, whereas non‐model microorganisms have received much less attention in this regard. The floral nectar of angiosperms is an ephemeral, island‐like habitat for different highly adapted yeasts and bacteria. The growth of microorganisms in floral nectar depends on their ability to efficiently use the available nutrients and tolerate challenging physicochemical conditions, including high osmotic pressures, unbalanced carbon‐to‐nitrogen ratios, and the presence of diverse defensive compounds of plant origin. The production of alternative phenotypic states in response to environmental cues (i.e., phenotypic plasticity) or independently from these (within‐environment trait variability) might be particularly relevant in floral nectar, in which rapid growth is needed for population persistence and to improve the chance of animal‐mediated dispersal. In this article, we use the nectar microbiome as an example to encourage further research on the causes and ecological consequences of phenotypic plasticity and within‐environment trait variability of microbes. We review previous work on the mechanisms and potential ecological significance of the phenotypic plasticity and within‐environment trait variability displayed by nectar yeasts and bacteria. Additionally, we provide an overview of some topics that require further attention, including potential trade‐offs between different traits that are relevant for adaptation to dynamic nectar environments and the direct and indirect effects of phenotypic variability on the fitness of plants, flower‐visiting animals, and other nectar microbes. We conclude that further research on the causes and ecological consequences of phenotypic plasticity and within‐environment trait variability of microbes is essential to get a better understanding of community assembly and the establishment of ecological interactions in floral nectar and other similar highly dynamic and strongly selective microbial habitats.

## Graphical abstract

We use the floral nectar microbiome of angiosperms as an example to encourage further research on the causes and ecological consequences of phenotypic plasticity and within‐environment trait variability of microbes. We review previous work on the mechanisms and potential ecological significance of the phenotypic plasticity and within‐environment trait variability displayed by nectar yeasts and bacteria and provide an overview of some topics that require further attention, including the direct and indirect effects of phenotypic variability on the fitness of plants, flower‐visiting animals, and other nectar microbes.

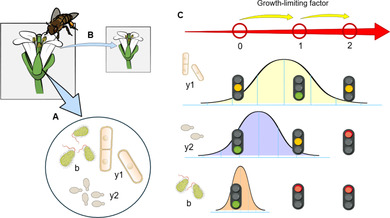

## 1 Introduction

Understanding the rules that govern the relationship between phenotypic diversity, genetic structure, and the ecological success of individuals, populations, and species is a common theme in evolutionary biology (Forsman 2015; Fusco and Minelli 2010; Gomulkiewicz and Stinchcombe 2022). Plastic responses, which may involve changes in morphological, physiological, developmental, and/or behavioral traits, allow individuals to match their phenotypes, or those of their offspring, to spatial and/or temporal variations in their abiotic and biotic environments (Forsman 2015; Fusco and Minelli 2010; Gomulkiewicz and Stinchcombe 2022; Reed et al. 2010).

Environmentally sensitive production of alternative phenotypes by individual genotypes, generally referred to as phenotypic plasticity (DeWitt and Scheiner 2004; Fox et al. 2019; Fusco and Minelli 2010), plays a key role in modulating how environmental variation influences population dynamics and community assembly in different ecosystems (Gómez et al. 2023; Pérez‐Ramos et al. 2019; Reed et al. 2010). Additionally, within‐environment trait variation, which typically refers to phenotypic variation within a given set of environmental conditions arising from genetic differences among individuals within the population, microenvironmental effects, and developmental instability, is also an important component of adaptive responses (Matesanz et al. 2021; Ørsted et al. 2018).

Most previous research on the causes, consequences, and costs of both phenotypic plasticity and within‐environment trait variation has focused on animals and plants (see Agrawal 2001; Borges 2008; Palacio‐López et al. 2015 for some comprehensive reviews). Previous work on these topics has also made use of a few microbial systems, including the baker's yeast ( *Saccharomyces cerevisiae* ) (e.g., Duveau et al. 2017; Peltier et al. 2018) and bacterial species such as *Escherichia coli* (e.g., Fraebel et al. 2020; Friesen et al. 2004) and *Pseudomonas aeruginosa* (e.g., Kümmerli et al. 2009). In contrast, the study of phenotypic plasticity and genotype–phenotype‐habitat associations in non‐model microorganisms has traditionally lagged behind. Nevertheless, the realization that microorganisms represent a vast untapped reservoir of genetic and metabolic diversity that is key to the functioning of the biosphere (Escalas et al. 2019; Hurst 2021; Prosser et al. 2007) and the technological advances allowing the cultivation and high‐throughput phenotyping of understudied microbial lineages (Acin‐Albiac et al. 2020; Bochner 2009) have led to a renewed interest in understanding the role that phenotypic variation has on the assembly and dynamics of microbial populations and communities (D'Souza 2020; Krause et al. 2014; van Boxtel et al. 2017).

With this article, we aim to show that, as observed in many plant and animal communities, phenotypic plasticity and within‐environment trait variability might be key for habitat adaptation and community assembly in microbial ecosystems. To do so, we will focus on the microbial communities inhabiting the floral nectar of angiosperms, due to the increasing interest in using these communities to study diverse ecological questions related to community assembly, their crucial involvement in plant–pollinator interactions, and their intrinsic properties that make them interesting for the study of phenotypic plasticity (see Section 2). Additionally, we discuss some knowledge gaps about the ecological significance of phenotypic plasticity and within‐environment trait variability of nectar microbes, including potential trade‐offs between different traits allowing persistence in dynamic nectar environments and the potential downstream effects of phenotypic variability on plant–animal mutualisms and microbe–microbe interaction networks.

## 2 Floral Nectar as a Model System for the Study of Phenotypic Plasticity

Floral nectar is a quintessential example of highly variable microenvironment at different spatial and temporal scales (Nicolson 2022; Nicolson and Thornburg 2007; Parachnowitsch et al. 2019). Beyond its crucial role in plant–pollinator interactions, this sugary secretion of flowers has begun to stand out as a natural habitat for many kinds of microorganisms, and more specifically unicellular fungi (also known as yeasts) and bacteria, that ultimately can influence the mutualistic relationships between angiosperms and their animal visitors (Barberis et al. 2024; Jacquemyn et al. 2021; Pozo, Lievens, et al. 2015; Vannette 2020).

**FIGURE 1:**
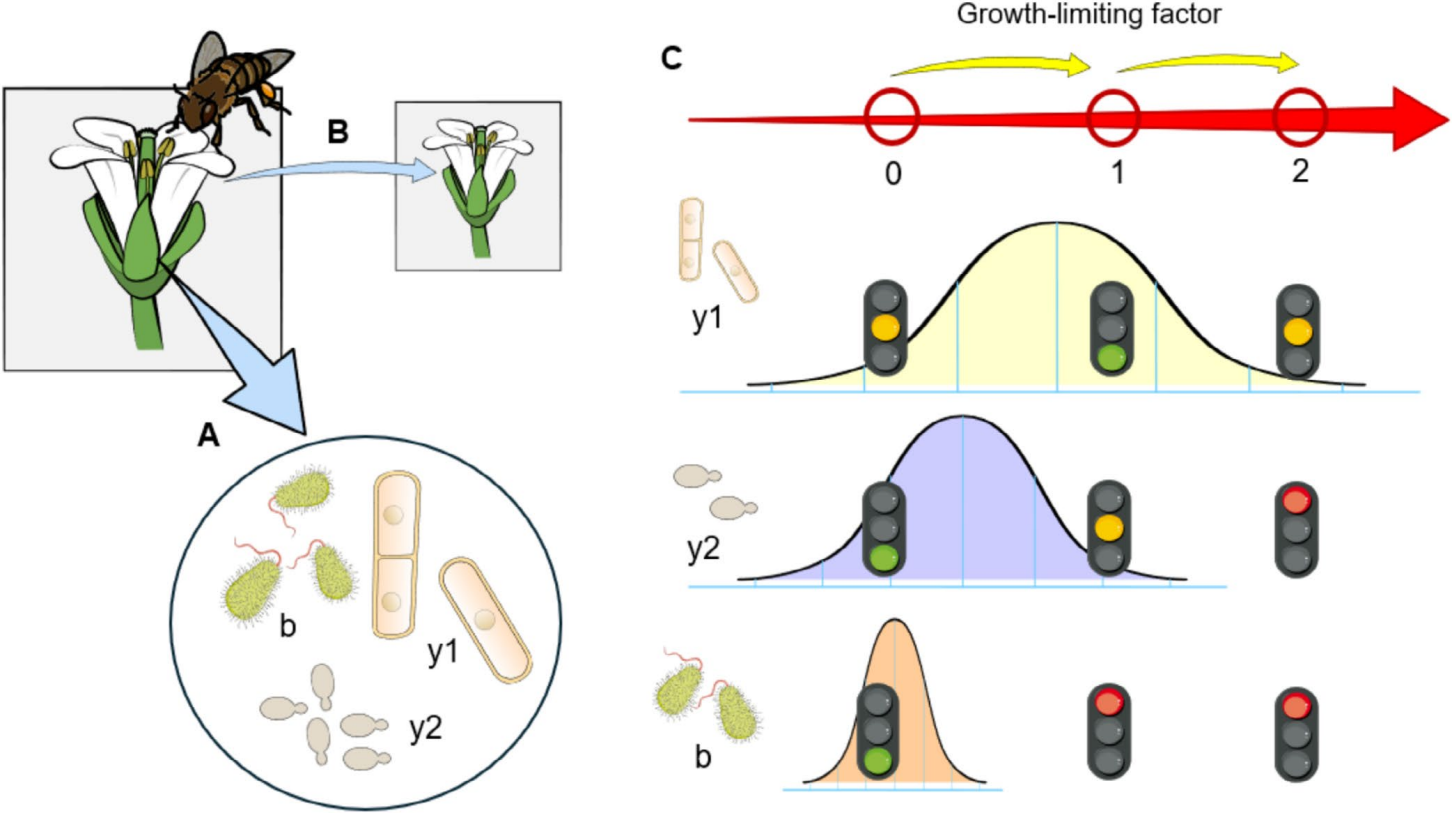
Phenotypic plasticity as a potential driver of habitat adaptation and community assembly in the floral nectar microbiome. The floral nectar of angiosperms is a complex solution of sugars, proteins, amino acids, minerals, and other components (e.g., secondary metabolites and volatile organic compounds) that plays a crucial role in pollinator attraction and other interactions with animals. Floral nectar is also the natural habitat of diverse microorganisms (A), including yeasts (y1 and y2) and bacteria (b). Rapid growth is key for microbial population persistence in this habitat and to increase the chance of animal‐mediated dispersal (B). Phenotypic plasticity (i.e., environmentally sensitive production of alternative phenotypes by individual genotypes) might also be key for survival in nectar (C), as those species with a broader tolerance (y1 > y2 > b) to sudden changes in growth‐limiting factors (from state 0 to state 1, and then to state 2) will grow better (green traffic light), or at least survive better (e.g., suboptimal growth or persistence in a quiescent state; amber light), than the other species (red light). In this scenario, the combination of environmental variability and differences in phenotypic plasticity will result in different community composition (y1 + y2 + b in state 0, y1 + y2 in state 1, and y1 in state 2). Figure created with Bioicons (https://bioicons.com).

Growth of microorganisms in floral nectar depends on their capacity to efficiently use the available nutrients and to tolerate high osmotic pressures, unbalanced carbon‐to‐nitrogen ratios, and the frequent presence of diverse toxins of plant origin (Herrera 2017; Herrera et al. 2010; Lievens et al. 2015; Pozo and Jacquemyn 2019; Pozo et al. 2012). Besides, flowers are ephemeral, island‐like habitats in which rapid growth is needed for population persistence and to improve the chance of animal‐mediated dispersal (Belisle et al. 2012; Crowley and Russell 2021; Hausmann et al. 2017). Therefore, it can be hypothesized that phenotypic plasticity, which allows organisms to rapidly switch between different phenotypic states in response to environmental cues and may enable them to overcome habitat filters (e.g., Gianoli and Escobedo 2021; Mallard et al. 2020), might represent a major factor driving habitat adaptation and microbial community assembly in floral nectar (Figure 1).

The short generation times of most species of nectar microbes, the relative simplicity of nectar microbial communities compared to other natural microbiomes (e.g., rhizosphere and phyllosphere), and their organization in a well‐defined hierarchical structure of increasing complexity (nectaries within flowers, flowers within individual plants, plants within populations, etc.) that allows multi‐scale approaches have contributed to the growing interest in using the floral nectar microbiota as a model system for the study of various ecological processes, such as competitive exclusion, dispersal, historical contingency, and metacommunity dynamics (Chappell and Fukami 2018; Klaps et al. 2020; Quevedo‐Caraballo et al. 2025). Use of nectar microorganisms as model systems for the study of phenotypic plasticity and its ecological consequences might offer similar advantages, but this possibility remains largely underexplored. It is important to note that, despite the technological progress of recent years, the phenotypic and functional study of microorganisms, and of nectar microbes in particular, remains challenging and faces some major limitations (Table 1). Among these, although single‐cell metabolic profiling and phenotypic screening of microorganisms are already possible (Chen et al. 2022), these are still usually studied at a population level, assuming that bulk‐cell assays describe the average status of individuals in the population. In addition, while most attention is still paid to traits related to microbial growth (e.g., cell density, viable counts, colony size, growth rate, etc.) and resource use (e.g., nutrient consumption or metabolite production), variability in many other traits that might improve fitness in highly fluctuating environments, such as the ability to persist until the biotic and/or abiotic conditions improve or to disseminate actively and/or passively to other habitat patches, is often ignored. Nevertheless, significant progress has been made in the last few years in analyzing the inter‐ and intra‐species variability of nectar microbes in some key traits favoring survival in their natural habitat, determining the link between the (phylo)genetic and phenotypic diversity of these microorganisms, and shedding light on the role of trait variability in the assembly of the nectar microbiome.

**TABLE 1:** Main challenges of studying the phenotypic and functional diversity of microorganisms, and nectar microbes in particular.

Challenges^a^	Peculiarities of nectar microbes
Most environmentally important microbes have yet to be cultivated, and most phenotypes can only be validated using culturable taxa. Transcriptomics, metagenomics, and other “‐omic” sciences are key to the phenotypic/functional study of non‐culturable microbes.	The transcriptomic and metabolomic study of nectar microbes is still in its infancy (but see Morris et al. (2020) and Chappell et al. (2024) for some pioneer studies).
The dormant component of microbial communities represents a vast reservoir of genetic and phenotypic diversity.	The dormant component of nectar microbial communities has not been studied yet, although *Metschnikowia reukaufii* (Ascomycota) and other nectar yeasts can produce chlamydospores.
Phenotypes should be measurable at the individual level, which is technically challenging for microorganisms. Single‐cell metabolic and phenotypic profiling of microorganisms is already possible, but most phenotypes are still usually studied at the population‐level	Single‐cell phenotyping and/or metabolic profiling has not yet been attempted for nectar microbes. Most published studies only rely on population‐level phenotypes.
Defining microbial species is controversial due to the lack of sexual reproduction in most cases, limited resolution power of universal gene barcodes used in microbial taxonomy, and horizontal gene transfer. This might affect the delineation of phenotypic classes.	Intense taxonomic work on nectar microbes has led to the description of new taxa of yeasts and bacteria. In some cases, (sub)species boundaries have been found to be fuzzy (e.g., Álvarez‐Pérez et al. 2023). Furthermore, evidence of genome mosaicism has been found for some nectar microbes (e.g., Álvarez‐Pérez, Dhami, et al. 2021).
Selecting and measuring microbial traits that are relevant to community assembly and ecosystem function is a difficult task.	Most phenotypic studies of nectar microbes have focused on traits related to growth performance (e.g., cell density, viable counts, colony size, and growth rate) and resource use, overlooking other traits that might improve fitness in floral nectar.
Microorganisms often respond to changes in their environment through phenotypic plasticity in multiple traits (i.e., multivariate plasticity) that can interact and affect each other's development and selection.	Most nectars are characterized by high sugar and low nitrogen content, so the response of nectar microbes to increased osmotic pressure and nitrogen limitation cannot be uncoupled (Morales‐Poole et al. 2023). Additionally, nectar microbes must deal with the frequent presence in floral nectar of hydrogen peroxide, alkaloids, cardenolides, terpenoids, and other toxic compounds of plant origin (Landucci and Vannette 2024; Mueller et al. 2023; Vannette and Fukami 2016).

^a^ References: Chen et al. (2022), Escalas et al. (2019), Nemergut et al. (2013), Nielsen and Papaj (2022), Prosser et al. (2007).

## 3 Phenotypic Variability of Nectar Microbes: Evidence and Ecological Significance

Nectar microbial communities typically function as metacommunities (i.e., sets of local communities that are connected to each other by occasional dispersal) with high patch turnover rates and intense colonization–extinction dynamics (Chappell and Fukami 2018; Jacquemyn et al. 2021; Pozo, Lievens, et al. 2015). Nectar chemistry can vary widely within and between the different plant species in which microbes become inoculated (Herrera et al. 2006; Nicolson and Thornburg 2007; Palmer‐Young et al. 2019), so growth in this floral microhabitat depends on the ability of microorganisms to rapidly adapt to sometimes extremely different types of nectar. As indicated above (Figure 1), phenotypic plasticity might be a major driving factor of habitat adaptation and community assembly in floral nectar. In this section, we present different lines of evidence suggesting that phenotypic plasticity and within‐environment trait variability might be important attributes for the ecological success of nectar microbes.

### 3.1 Nectar Microbes Explore Wide Phenotypic Landscapes

Variation in traits of individuals and species, especially those involved in resource allocation and stress tolerance, is essential to understand and predict species interactions and community composition (Berg and Ellers 2010). Resource requirement and resource uptake are plastic traits that can alter the fundamental niche (i.e., the range of environmental factors under which a species can survive, grow, and reproduce in the absence of other species) and the realized niche (i.e., the part of the fundamental niche of a species that this occupies in presence of other species) of the members of a community when environmental conditions change (Berg and Ellers 2010).

Most phenotypic studies of nectar microbes have dealt with a few species of nectar specialists, including the yeasts of genus *Metschnikowia* (Ascomycota). Species such as *Metschnikowia reukaufii* and *Metschnikowia gruessii*, which are highly prevalent in the floral nectar of phylogenetically diverse plants in different biomes (Álvarez‐Pérez and Herrera 2013; Belisle et al. 2012; Brysch‐Herzberg 2004; Pozo et al. 2011), seem to reproduce mostly clonally in nature but exhibit, nevertheless, high intra‐species genetic diversity (Álvarez‐Pérez, Dhami, et al. 2021; Dhami et al. 2018; Herrera et al. 2011, 2014). Furthermore, *M. reukaufii* and *M. gruessii* populations associated with insect‐pollinated plants in southeastern Spain show remarkable host‐mediated genetic diversification, with genotypes being non‐randomly distributed among flowers of different plant species or flowers of conspecific individuals locally co‐occurring (Herrera et al. 2011, 2014). In contrast, no geographic or host‐dependent genetic or phenotypic clustering was found by Dhami et al. (2018) for *M. reukaufii* populations associated with the hummingbird‐pollinated shrub *Diplacus (Mimulus) aurantiacus* (Phrymaceae) in California, USA.

Leaving aside the genetic makeup of wild yeast populations, it has been shown that both *M. reukaufii* and *M. gruessii* can explore wide phenotypic landscapes that include traits related to the assimilation of multiple nutrients and tolerance to diverse growth‐inhibiting conditions frequently encountered in floral nectar (e.g., osmotic stress) (Álvarez‐Pérez, Dhami, et al. 2021; Dhami et al. 2018; Pozo et al. 2012, 2016; Pozo, Herrera, et al. 2015). Variation in such traits seems to be linked to the plant species from which yeast isolates are obtained (Pozo, Herrera, et al. 2015). Additionally, *Metschnikowia* spp. and other nectar yeasts display inter‐ and intra‐species variability in their tolerance to plant defensive chemicals (Mueller et al. 2023; Pozo et al. 2012, 2016; Pozo, Herrera, et al. 2015) and to 14α‐demethylase inhibitors and other fungicides of common use in agriculture whose residues can disperse and persevere in the environment (Álvarez‐Pérez et al. 2016; Bartlewicz et al. 2016; Quevedo‐Caraballo et al. 2024). This latter characteristic might be a relevant selective force in wild plant communities located near crop fields. However, there is still very little information on the short‐ and long‐term effects of exposure to fungicides and other anthropogenic pollutants on nectar yeast populations.

High phenotypic diversity in relation to nutrient acquisition and tolerance to different stressors has also been observed for nectar specialist bacteria but, again, published results mostly refer to members of the Gammaproteobacteria class (Pseudomonadota), including the *Acinetobacter nectaris–Acinetobacter boissieri* clade and genus *Rosenbergiella* (Álvarez‐Pérez, Baker, et al. 2021; Álvarez‐Pérez et al. 2023; Álvarez‐Pérez, Tsuji, et al. 2021; Lenaerts et al. 2014; Morales‐Poole et al. 2023).

The wide phenotypic landscapes explored by nectar microbes might help them to cope with a vast array of nectar physicochemical conditions. However, it is important to consider that microbial phenotypes are generally tested using standardized in vitro assays that are often radically different from the conditions met by nectar microbes in nature, where environmental heterogeneity is oversimplified and the only limiting factor is the element being tested. For example, although it is well known that nectar chemistry varies widely across plant species and even between different flowers of the same species or nectaries of the same flower (Herrera et al. 2006; Nicolson and Thornburg 2007), this variation in nectar physicochemical characteristics is rarely considered in phenotypic studies of nectar microbes. Moreover, most published studies overlook that organisms often respond to changes in their environment through multivariate plasticity (but see, for example, Morales‐Poole et al. (2023) and Vannette and Fukami (2014) for analysis of microbial growth responses and community assembly dynamics, respectively, in floral nectars differing in multiple characteristics). Besides, for the sake of simplification, experiments assessing the growth performance of nectar microbes in different conditions typically include cells of a single yeast or bacterial isolate, ignoring that different microbial species and/or genotypes of the same species frequently co‐occur in floral nectar (Álvarez‐Pérez and Herrera 2013; Cecala et al. 2024; Sharaby et al. 2020). This experimental approach neglects the possible role of microbe–microbe interactions in determining community assembly and trait variability in nectar (Álvarez‐Pérez et al. 2019). A greater focus should also be placed on the speed at which phenotypic changes occur (i.e., rate of plasticity), as it directly relates to the adaptiveness of plastic responses (Burton et al. 2022; Dupont et al. 2024). Nectar microbes typically undergo a combination of osmotic and nutritive stress (Morales‐Poole et al. 2023), so the speed at which the enzymes and other proteins involved in the response to these processes are produced might determine the survival of individual species and the interaction of these with other members of the community. Finally, nectar yeasts and bacteria might overcome environmental harshness by entering a quiescent state and then resuming growth when environmental conditions improve (e.g., dilution of nectar by further secretion, arrival of nutrient inputs from external sources, etc.). In this regard, it has been observed that *M. reukaufii* often produces chlamydospores, which are enlarged, thick‐walled cells that might allow persistence under environmental stress (Dhami et al. 2018; Lachance 2016). Bacteria can also produce endospores and other dormant cell forms that allow them to survive in stressful environments (Niu et al. 2024), but the prevalence and ecological relevance of these bacterial “persisters” remain to be studied in floral nectar.

### 3.2 Ecologically Relevant Traits of Nectar Microbes Are Predicted by Phylogeny

A common expectation in trait biology is that close phylogenetic relatives phenotypically resemble each other more than distant lineages (Goberna and Verdú 2016; Kamilar and Cooper 2013; Martiny et al. 2015). Different studies have revealed some correlation between phylogeny and ecologically relevant traits in nectar microbes. For example, Álvarez‐Pérez, Tsuji, et al. (2021) demonstrated that phylogenetic relatedness between *A. nectaris/A. boissieri* clade members was associated with their ability to assimilate diverse amino acids and other nitrogen sources commonly found in floral nectar. Similarly, Morales‐Poole et al. (2023) detected phylogenetic signal in the growth performance of *Acinetobacter* spp. and *Rosenbergiella* spp. in a series of synthetic nectars differing in their overall sugar content, overall nitrogen content, and sugar composition. Nevertheless, the phylogenetic signal of most traits analyzed in these studies were found to be very shallow and to rapidly vanish above the species level (Álvarez‐Pérez, Tsuji, et al. 2021; Morales‐Poole et al. 2023).

The link between phylogenetic affiliation and trait resemblance has also been studied for nectar yeasts. Herrera et al. (2010) demonstrated that the yeast communities inhabiting the floral nectar of bumble bee‐pollinated stinky hellebores ( *Helleborus foetidus* , Ranunculaceae) in Spain were phylogenetically biased with respect to the species pool found on *Bombus terrestris* (Hymenoptera: Apidae) glossae, being enriched in osmotolerant members of the *Metschnikowiaceae* family (*M. reukaufii*, *M. gruessii*, and *Metschnikowia (Candida) rancensis*). Using microcosm experiments, Peay et al. (2012) found considerable variation in the strength of priority effects (i.e., a type of historical contingency in which the effects of species on one another depend on their arrival order into a local site and initial abundance; Stroud et al. 2024) between nectar yeasts depending on their phylogenetic relatedness. Specifically, the competitive exclusion between early‐ and late‐arriving yeast species was stronger when these were closely related. Moreover, these authors found a correlation between phylogenetic relatedness and physiological similarity among nectar yeasts (growth characteristics in different media and consumption of different sugars and amino acids), with the latter being considered a proxy for ecological similarity (Peay et al. 2012). At the intra‐species level, Dhami et al. (2018) observed that the delineation of genomic lineages of *M. reukaufii* found in the nectar of *D. aurantiacus* correlated well with differences in the growth performance of isolates in permissive and restrictive media, their patterns of nutrient utilization, and their ability to compete against *M. rancensis*. In contrast, a subsequent study in southeastern Spain revealed high phenotypic overlap between genomic populations of *M. reukaufii* and weak phylogenetic signal for the assimilation of different nutrient sources and tolerance inhibitors (Álvarez‐Pérez, Dhami, et al. 2021). Differences in the study level (inter‐ vs. intra‐species), phenotypic traits analyzed (e.g., biological interactions vs. physiological performance), and sampling design (number of strains, geographic origin, plant hosts, etc.) might explain the contrasting results between these studies.

Most previous studies testing for phylogenetic signal in different traits of nectar microbes suffer from two major limitations: (i) they were based on a selection of yeast or bacterial isolates from a limited number of plants (or their pollinators) and geographical regions, which precludes any generalization about the relative role of phylogenetic relatedness over habitat and/or geographical factors in determining microbial phenotypes; and (ii) phylogenetic determination was evaluated using average trait values calculated from different replicates of the same experimental condition, instead of the variance or any other measure of dispersion that might be more informative of within‐environment variability of key adaptive traits. Similarly, the results of these studies might be affected by convergent evolution, genetic recombination, horizontal gene transfer, and other genetic and non‐genetic factors (see Section 3.3) that can blur the phylogenetic signal of traits (Álvarez‐Pérez, Dhami, et al. 2021; Goberna and Verdú 2016; Kamilar and Cooper 2013).

### 3.3 Trait Variability of Nectar Microbes Is Genetically and Epigenetically Determined

The genetic and non‐genetic determinants of phenotypic plasticity have been the object of abundant research (Kovuri et al. 2023; Vogt 2023). Among the genetic factors involved, recombination due to (para)sexual reproduction is a major cause of genetic diversity that can accelerate exploration of new phenotypes, although it may also disrupt well‐adapted phenotypes previously existing in the population (Hu et al. 2014; Wagner 2011). Sexual reproduction seems to be rare in the nectar yeast populations studied to date, but it has been hypothesized that *Metschnikowia* species might occasionally mate in other habitats (e.g., in bumble bee nests) and result in some genetic recombination (Herrera et al. 2011, 2014). Genome mosaicism seems to be relatively common in some *M. reukaufii* populations, and it has been suggested that genetic admixture might contribute to the adaptation of nectar yeasts to highly variable nectar environments via transgressive evolution (Álvarez‐Pérez, Dhami, et al. 2021; Dhami et al. 2018).

Even in the absence of sexual recombination in most species of nectar microbes, phenotypic plasticity can stem from mutation, horizontal gene transfer, and other genetic factors. Non‐deleterious and non‐neutral mutations are fundamental for the generation of phenotypic diversity, especially when they occur in “hub loci” that intensify or buffer the phenotypic manifestation of other loci and have therefore a higher potential to drive evolutionary change (Kovuri et al. 2023; Vogt 2023). The limited genomic research carried out so far with nectar microbes has revealed that, through tandem duplications of genes involved in nitrogen metabolism and transport (e.g., some high‐capacity amino acid importers that are homologs of *S. cerevisiae* general amino acid permease1 [*GAP1*] and proline utilization 4 permease [*PUT4*]), the nectar yeast *M. reukaufii* has evolved strategies for efficient nitrogen scavenging (Dhami et al. 2016). The overexpression of these genes under nitrogen‐limiting conditions might allow *M. reukaufii* to thrive under conditions where other microbes will perish (Dhami et al. 2016). Regarding horizontal gene transfer, viral sequences have been found in the genome of nectar specialist bacteria such as *A. nectaris* and *Rosenbergiella nectarea* (Laviad‐Shitrit et al. 2020; Sanchez et al. 2025). Additionally, Sanchez et al. (2025) have recently suggested that the *A. nectaris/A. boissieri* clade might have acquired pectin degrading enzymes through horizontal gene transfer from necrotrophic plant pathogens in the order *Enterobacterales* (Pseudomonadota: Gammaproteobacteria) such as *Pectobacterium*, *Erwinia*, and *Dickeya*, and that this genome innovation might allow the breakdown of pollen grains that fall into nectaries and be essential to overcoming nutrient limitation in this habitat. Horizontal gene transfer can also happen in eukaryotes (Keeling 2024), but no proof of its occurrence has been found yet in nectar yeasts.

**FIGURE A1:**
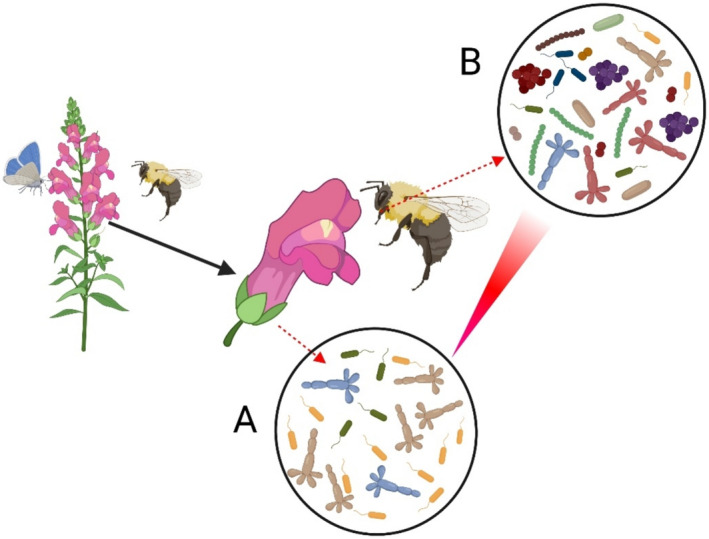
Species filtering and selective population bottlenecks in floral nectar. Nectar yeasts and bacteria depend on pollinators and other animal visitors of flowers for their dispersal. However, nectar microbial communities (A) are typically biased with respect to the species pool found on the body of their dispersal agents (B), being enriched in yeast and bacterial species (represented in A and B by different shapes) that can overcome the challenging conditions for microbial growth of floral nectar (e.g., increased osmotic pressure due to high sugar content, nitrogen limitation, and presence of defensive compounds of plant origin). Besides, at the intra‐species level, different phenotypes (represented in A and B by different colors) might also differ in their ability to use nectar's nutrient resources and tolerate the presence of growth inhibitors, so selective population bottlenecks might not be uncommon in nectar microbial communities. Figure created with BioRender: Álvarez‐Pérez, S. (2024). http://biorender.com/j13p894.

Genetic drift, that is, the random change in the frequency of an allele from generation to generation, may also influence the spectrum of phenotypic diversity in a population by either causing gene variants to disappear or initially rare alleles to become more frequent (Vogt 2023). Population bottlenecks leading to a dramatic reduction in population size, which are common in the evolutionary dynamics of natural microbial populations, are major causes of genetic drift, can result in a significant reduction in genetic diversity, and potentially determine microbial evolvability (Wein and Dagan 2019). As indicated above, nectar microbial communities are typically organized as metacommunities, and both nectar yeasts and bacteria depend predominantly on pollinators and other animal visitors of flowers for their dispersal from flower to flower (Brysch‐Herzberg 2004; Donald et al. 2022; Hausmann et al. 2017; Herrera et al. 2010; Vannette and Fukami 2017; Vannette et al. 2021). Therefore, population bottlenecks associated with dispersal might not be uncommon in nectar microbial communities. These bottlenecks could be non‐selective if all species or phenotypes of the same species were equally likely to survive, or selective if some species/phenotypes were more likely to survive than others (e.g., if habitat filtering occurs due to challenging nectar conditions) (Moxon and Kussell 2017). Selective bottlenecks due to habitat filtering seem to prevail in floral nectar and have relevant consequences for the fitness of nectar microbial populations and the assembly of the nectar microbiome (see Figure A1 in the Appendix A) (Herrera 2014; Herrera et al. 2010).

Apart from genetic factors, several non‐genetic factors can contribute to trait determination, including the spatiotemporal activity of cells (e.g., age, growth phase and molecular dynamics), intracellular stochasticity (e.g., due to uneven distribution of macromolecules or cell organelles during cell division), and epigenetic modifications (Kovuri et al. 2023). Of these non‐genetic factors, epigenetic changes such as DNA methylation have received the most attention as mechanisms enabling low‐cost exploration of phenotypic spaces and response to diverse environmental pressures (Santiago et al. 2022; Vogt 2023). Herrera et al. (2012) screened genome‐wide DNA methylation at 120 methylation‐sensitive amplified polymorphism (MSAP) markers in genotypically distinct strains of *M. reukaufii* that were grown on media differing in their nutrient composition. The results of this study demonstrated that sugar composition, sugar concentration, and their interaction significantly influenced the probability that MSAP markers changed from unmethylated to methylated. Moreover, the overall proportion of methylation was found to be related to the overall sugar concentration, with the lowest methylation rate occurring at 30% w/v and the highest at 50% w/v, and inhibiting methylation with the nucleoside analogue 5‐azacytidine compromised *M. reukaufii* growth in sugar‐containing media but had no detectable effect in control media (Herrera et al. 2012). These results clearly indicate that the phenotypic plasticity of resource use via flexible DNA methylation can become more important as environmental conditions harden, and elegantly connect environmentally‐driven within‐genotype epigenetic variation, phenotypic plasticity, and a conferred ecological advantage (niche width enhancement) to nectar yeast populations (Herrera et al. 2012; Schrey and Richards 2012).

Similarly, Sipiczki and Czentye (2024) have recently described a reversible stochastic epigenetic‐like silencing of the production of pulcherrimin (i.e., red pigment consisting in a complex of pulcherriminic acid and ferric ions that immobilizes iron and adversely affects the growth of other microorganisms) in different members of the *Metschnikowia pulcherrima* clade, a generalist yeast group that is often found in floral nectar but thrives in other floral and non‐floral habitats (Quevedo‐Caraballo et al. 2024). Notably, such stochastic production of pulcherrimin results in some form of within‐environment trait variation, as *M. pulcherrima* colonies often display sectors with different pigmentation due to a differential secretion of the pigment (Sipiczki et al. 2024; Sipiczki and Czentye 2024). Notably, heavily pigmented clones of *M. pulcherrima* have higher inhibitory activity on the ascomycetous yeasts *Debaryomyces* aff. *hansenii* and *Zygosaccharomyces* aff. *siamensis* than non‐pigmented clones, thus linking phenotypic variability with the outcome of interspecies interaction in this yeast species (Sipiczki and Czentye 2024).

The importance of other epigenetic mechanisms, such as post‐translational histone modifications, non‐coding RNAs, mRNA editing, and mRNA modifications (Vogt 2023), in determining phenotypic plasticity and habitat adaptation in nectar yeasts remains unknown. Finally, epigenetic changes can also occur in prokaryotes (Oliveira 2021; Sánchez‐Romero and Casadesús 2020), but their prevalence in the bacterial inhabitants of floral nectar has not been studied yet.

### 3.4 Trait Variability Might Allow Resource Partitioning and Nutritional Interaction Between Nectar Microbes

Partitioning of resources according to time, space, or resource properties is frequently observed between and within sympatric species (Suzuki and Arita 2014). Additionally, nutritional interactions can facilitate or hinder co‐occurrence patterns of different microbial species and/or genotypes in natural habitats (Bever et al. 2010; Kost et al. 2023). Notably, in many cases, resource partitioning and nutritional interactions are trait‐mediated and depend on the phenotypic changes of the interacting individuals (Agrawal 2001; Suzuki and Arita 2014).

Pozo et al. (2016) tested the prediction that niche differentiation explained the frequent co‐occurrence between *M. reukaufii* and *M. gruessii* in the floral nectar of diverse plants in Southern Spain and observed that both yeast species displayed significantly different physiological profiles and might not compete for most carbon and nutrient sources in nectar. Moreover, *M. reukaufii* and *M. gruessii* also seem to differ in their phenotypic response to variation in nectar's physicochemical conditions (e.g., by modifying the availability of different sugar sources or adding diverse growth inhibitors). Interestingly, at least under some nectar conditions, facilitation of growth between these two yeast species prevails over competition (Pozo et al. 2016).

Resource partitioning has also been suggested to occur between nectar bacteria and yeasts. Some early studies on nectar microbial communities found non‐random co‐occurrence between *A. nectaris/A. boissieri* clade members and cosmopolitan nectar yeast specialists such as *M. reukaufii* and *M. gruessii* in the floral nectar of Mediterranean plants from Spain (Álvarez‐Pérez and Herrera 2013). Then, it was found that *Metschnikowia* spp. and *A. nectaris*/*A. boissieri* have complementary carbon assimilation profiles, with the yeasts preferentially using glucose and enriching floral nectar in fructose and the bacteria mostly feeding on fructose (Álvarez‐Pérez et al. 2013; Álvarez‐Pérez, Baker, et al. 2021), and that the nectar acinetobacters can assimilate ammonium, urea, and different amino acids that are normally scarce in floral nectar but may be released by yeasts as metabolic by‐products (Álvarez‐Pérez, Tsuji, et al. 2021).

All in all, it might be postulated that resource partitioning is a major driver of yeast–yeast and yeast–bacterium interaction in the nectar microbiome. Although attractive, this hypothesis has some major flaws. For example, the presumed positive co‐occurrence between nectar yeasts and bacteria has been questioned by some recent studies (e.g., de Vega et al. 2021; Chappell et al. 2022) and the suggested nutrient resource partitioning between *Metschnikowia* and *Acinetobacter* spp. remains to be experimentally tested. Besides, not all yeast and bacterial strains have the same ability to feed on different nutrient sources (Álvarez‐Pérez, Dhami, et al. 2021; Álvarez‐Pérez, Tsuji, et al. 2021; Dhami et al. 2018; Morales‐Poole et al. 2023; Pozo et al. 2016; Pozo, Herrera, et al. 2015), so resource partitioning might be contingent on the genetic background of the interacting partners. Additionally, *M. reukaufii* is an efficient scavenger of nitrogen that can adapt its phenotype in nutrient‐limiting conditions (Dhami et al. 2016), so it remains uncertain whether competition between nectar microbes is more prevalent than resource partitioning or facilitation in floral nectar (Álvarez‐Pérez, Tsuji, et al. 2021). Finally, to our knowledge, the possible occurrence of resource partitioning between isogenic (i.e., genetically uniform) cells in homogeneous environments, such as that observed between *E. coli* populations with initially genetically identical individuals (San Roman and Wagner 2018), has not been demonstrated yet for any species of nectar microbe.

### 3.5 Trait Variability Can Alter Priority Effects Between Nectar Microbes

Nectar microbial communities are powerful systems to study the different factors affecting community assembly (Chappell and Fukami 2018; Klaps et al. 2020). The interaction between phenotypic plasticity and community assembly in the nectar microbiome has been studied in less detail; however, for example, there is some evidence suggesting that trait variability might contribute to overcoming priority effects.

Using microcosm experiments, Tucker and Fukami (2014) demonstrated that the differential ability of *M. reukaufii* and bacteria from the genus *Neokomagataea* (Pseudomonadota: Alphaproteobacteria) to adapt their growth rate to variable vs. constant temperatures might blur priority effects and allow their coexistence in nectar communities. Furthermore, *M. reukaufii* can rapidly evolve resistance against the negative priority effect due to *A. nectaris*‐induced acidification of nectar (Chappell et al. 2022). At the molecular level, Chappell et al. (2024) analyzed the whole transcriptome of 108 *M. reukaufii* genotypes and detected several genes that were differentially expressed in synthetic nectars that had previously supported the growth of *M. rancensis*, which simulated the effect of niche preemption. In particular, exposure to *M. rancensis*‐conditioned nectar altered the expression of genes involved in amino acid metabolism, therefore suggesting that competition is an important mechanism of priority effects between nectar yeasts (Chappell et al. 2024). Additionally, genotypic identity greatly influenced *M. reukaufii* transcriptomic response to nectar conditions, thus highlighting that the interplay between genotype and environment is necessary to predict the mechanisms of microbe–microbe interaction in the nectar microbiome (Chappell et al. 2024).

## 4 Knowledge Gaps

All the evidence presented above supports that phenotypic plasticity is common in nectar microbial populations, that it can contribute to the adaptation of nectar microbes to the challenging environment of floral nectar, and that it might determine the assembly of the nectar microbiome. However, there are some major knowledge gaps about the potential trade‐offs between traits that are relevant for microbial survival in dynamic nectar environments and the effects that the phenotypic variability of nectar microbes might have on plant‐microbe‐animal and microbe–microbe interactions.

### 4.1 Phenotypic Trade‐Offs in Nectar Microbes

The costs and benefits of phenotypic plasticity under different ecological scenarios have been the object of intense discussion (DeWitt et al. 1998; DeWitt and Scheiner 2004; Van Buskirk and Steiner 2009). Different lines of evidence suggest that greater phenotypic plasticity should be favored in scenarios where spatial and temporal variation in environmental conditions is greater and dispersal is higher (Hendry 2016), which coincides with the prevailing conditions faced by nectar microbes. Therefore, phenotypic plasticity might likely contribute to the adaptation of nectar microbes to ephemeral and highly variable habitat patches.

Microbial fitness is typically measured in terms of growth performance in comparison to a reference condition and/or to other genotypes of the same species or different species (Fink and Manhart 2024; Pope et al. 2010). Maximization of growth rate is an important fitness strategy for microbes, but in many circumstances there exist trade‐offs between growth and other relevant traits, such as adaptability and survival, that could improve fitness in fluctuating environments and for which microorganisms need to adopt bet‐hedging strategies (Zhu and Dai 2024). Due to high inter‐ and intra‐plant variation in nectar nutritive quality and other physicochemical characteristics (Canto et al. 2007; Herrera et al. 2006; Liu et al. 2024; Palmer‐Young et al. 2019), the nutritional and microenvironmental conditions in nectar metacommunities are often highly fluctuating, which could lead to “feast and famine” cycles of microbial growth. To adapt to these alternating beneficial and challenging conditions, nectar microbes might need to coordinate plasticity in several different traits, including cellular growth and division, nutrient uptake, and/or storage and production of quiescent states (Section 3.1). A detailed analysis of the sophisticated molecular and cellular strategies used by yeasts and bacteria to efficiently modulate resource allocation to achieve optimal growth in various environmental conditions (see, e.g., Nguyen et al. 2024; Scott and Hwa 2023; Zhu and Dai 2024) might help to elucidate the strategies allowing microbial growth in floral nectar.

### 4.2 Phenotypic Variability of Nectar Microbes and Plant‐Microbe–Animal Interactions

Microbial activity has profound effects on nectar's physicochemical conditions. Nectar yeasts and bacteria can reduce the concentration of sucrose and amino acids and decrease the sucrose‐to‐hexose ratio of nectar, thus altering the nutritional quality of this floral secretion (Herrera et al. 2008; Lenaerts et al. 2017; Vannette and Fukami 2018). Furthermore, microbial metabolism can lower nectar pH by several units, produce ethanol as a byproduct, and increase the concentration of diverse volatile organic compounds that elicit behavioral responses in insects and other floral visitors (Chappell et al. 2022; Good et al. 2014; Lenaerts et al. 2017; Rering et al. 2018, 2020; Schaeffer et al. 2019; Vannette and Fukami 2016, 2018; Vannette et al. 2013). Additionally, there is abundant evidence that microbial growth in floral nectar can affect the fitness and foraging decisions of insect and vertebrate visitors of flowers and indirectly alter plant reproduction (de Vega et al. 2022; Herrera et al. 2013; Schaeffer and Irwin 2014; Yang et al. 2019; Vannette et al. 2013). In contrast, no study has addressed the effects that the phenotypic variability displayed by nectar microbes might have on plant–microbe–animal interactions.

Variability in microbial traits related to nutrient uptake and the release of metabolic by‐products might amplify (or buffer, depending on the case) the spatial and temporal variation in the quality of nectar as a reward for pollinators, which would have significant consequences for animal foraging decisions and, therefore, potentially impact plant reproduction and fitness. Trait variability might also interfere with the metacommunity dynamics of the nectar microbiome, for example, by modulating dispersal rates across habitat patches due to a greater or lower attraction of pollinators. So far, these hypotheses remain untested. Additionally, the ecological outcome of microbial trait variability might depend on multiple biotic and abiotic factors, so it is difficult to predict the overall effects (e.g., greater or lower attraction of floral visitors, increased or decreased reproductive success for plants, higher or lower dispersal of microorganisms).

### 4.3 Phenotypic Plasticity and Microbe–Microbe Interactions in Floral Nectar

Microbial communities are extremely complex entities in which the growth and activities of their members are affected by those of other members of the community and a myriad of abiotic factors (Meroz et al. 2024). Accordingly, microorganisms can rapidly switch between different phenotypic states in response to their surrounding environment and the activity of other microbes (Harrington and Sanchez 2014). Changes in interaction dynamics and structure due to phenotypic plasticity may scale up and affect the ecological network in which the plastic species are embedded (Berg and Ellers 2010; Gómez et al. 2023).

As indicated above (see Sections 3.4 and 3.5), different mechanisms such as resource partitioning, nutritional interactions, and historical contingency, which are often dependent on plastic traits, might be responsible for species co‐occurrence or co‐exclusion and determine community assembly in the nectar microbiome. Other mechanisms of microbe‐microbe interaction that might shape the nectar microbiome, such as antibiosis and signaling‐based interactions, have been pointed out in previous reviews (e.g., Álvarez‐Pérez et al. 2019) but remain to be analyzed in more detail. For instance, a particular type of interaction that should receive a greater focus is intra‐ and inter‐species cooperation via the secretion of public goods (e.g., enzymes and metabolic byproducts), which is often linked to the emergence of “cheating phenotypes” that do not cooperate but profit from the benefits produced by the cooperating individuals (Harrington and Sanchez 2014). While cooperation is widespread among yeasts and bacteria, can result in the optimization of limiting nutrient resources, and has an important role in intra‐ and inter‐species interactions (Celiker and Gore 2012; Damore and Gore 2012; Harrington and Sanchez 2014), the occurrence of this phenomenon directly linked to phenotypic plasticity has never been studied in the nectar microbiome. A detailed analysis of the relative role of cooperation vs. cheating in determining the dynamics of nectar microbial populations might provide clues on the assembly rules of the nectar microbiome and the benefits and costs of phenotypic plasticity for these microorganisms.

## 5 Conclusion and Perspectives

Through this review article, we try to encourage further research on the causes and ecological consequences of phenotypic plasticity and within‐environment trait variability of microbes, as that might be paramount to a better understanding of habitat adaptation and community assembly in different microbial ecosystems. Although we have focused on floral nectar, there are many other examples in nature of highly dynamic habitats imposing strong selective forces on microbial growth, in which phenotypic plasticity and within‐environment variability might be key for the survival and perpetuation of microbial populations and the establishment of ecological interactions. Future research should be aimed at analyzing how phenotypic plasticity interacts with the different factors that determine the assembly of microbial communities, including habitat filtering, priority effects, dispersal, and metacommunity dynamics. Additionally, there is a clear knowledge gap about the trade‐offs and the direct and indirect effects of microbial trait variability on the fitness of plants, animals, and other “macroorganisms”. Further research on these topics will help to clarify the ecological relevance of phenotypic plasticity and within‐environment variability for microorganisms.

## Author Contributions

**Sergio Álvarez‐Pérez:** conceptualization (lead), funding acquisition (lead), supervision (lead), writing – original draft (lead), writing – review and editing (equal). **Sergio Quevedo‐Caraballo:** resources (lead), writing – original draft (supporting), writing – review and editing (equal).

## Conflicts of Interest

The authors declare no conflicts of interest.

## Data Availability

The authors have nothing to report.

## References

[ece371059-bib-0001] Acin‐Albiac, M. , P. Filannino , M. Gobbetti , and R. Di Cagno . 2020. “Microbial High Throughput Phenomics: The Potential of an Irreplaceable Omics.” Computational and Structural Biotechnology Journal 18: 2290–2299. 32994888 10.1016/j.csbj.2020.08.010 PMC7490730

[ece371059-bib-0002] Agrawal, A. A. 2001. “Phenotypic Plasticity in the Interactions and Evolution of Species.” Science 294: 321–326. 11598291 10.1126/science.1060701

[ece371059-bib-0003] Álvarez‐Pérez, S. , L. J. Baker , M. M. Morris , et al. 2021. “ *Acinetobacter pollinis* sp. Nov., *Acinetobacter baretiae* sp. Nov. and *Acinetobacter rathckeae* sp. Nov., Isolated From Floral Nectar and Honey Bees.” International Journal of Systematic and Evolutionary Microbiology 71: 004783. 10.1099/ijsem.0.004783 33970854

[ece371059-bib-0004] Álvarez‐Pérez, S. , C. de Vega , M. I. Pozo , et al. 2016. “Nectar Yeasts of the *Metschnikowia* Clade Are Highly Susceptible to Azole Antifungals Widely Used in Medicine and Agriculture.” FEMS Yeast Research 16: fov115. 26703195 10.1093/femsyr/fov115

[ece371059-bib-0005] Álvarez‐Pérez, S. , C. de Vega , K. Vanoirbeek , et al. 2023. “Phylogenomic Analysis of the Genus *Rosenbergiella* and Description of *Rosenbergiella gaditana* sp. Nov., *Rosenbergiella metrosideri* sp. Nov., *Rosenbergiella epipactidis* Subsp. *epipactidis* Subsp. Nov., *Rosenbergiella epipactidis* Subsp. *californiensis* Subsp. Nov., *Rosenbergiella epipactidis* Subsp. Japonicus Subsp. Nov., *Rosenbergiella nectarea* Subsp. *nectarea* Subsp. Nov. and *Rosenbergiella nectarea* Subsp. *apis* Subsp. Nov., Isolated From Floral Nectar and Insects.” International Journal of Systematic and Evolutionary Microbiology 73: 005777. 10.1099/ijsem.0.005777. 36884370

[ece371059-bib-0006] Álvarez‐Pérez, S. , M. K. Dhami , M. I. Pozo , et al. 2021. “Genetic Admixture Increases Phenotypic Diversity in the Nectar Yeast *Metschnikowia reukaufii* .” Fungal Ecology 49: 101016.

[ece371059-bib-0007] Álvarez‐Pérez, S. , and C. M. Herrera . 2013. “Composition, Richness and Nonrandom Assembly of Culturable Bacterial‐Microfungal Communities in Floral Nectar of Mediterranean Plants.” FEMS Microbiology Ecology 83: 685–699. 23057414 10.1111/1574-6941.12027

[ece371059-bib-0008] Álvarez‐Pérez, S. , B. Lievens , and T. Fukami . 2019. “Yeast‐Bacterium Interactions: The Next Frontier in Nectar Research.” Trends in Plant Science 24: 393–401. 30792076 10.1016/j.tplants.2019.01.012

[ece371059-bib-0009] Álvarez‐Pérez, S. , B. Lievens , H. Jacquemyn , and C. M. Herrera . 2013. “ *Acinetobacter nectaris* sp. Nov. and *Acinetobacter boissieri* sp. Nov., Isolated From Floral Nectar of Wild Mediterranean Insect‐Pollinated Plants.” International Journal of Systematic and Evolutionary Microbiology 63: 1532–1539. 22904213 10.1099/ijs.0.043489-0

[ece371059-bib-0010] Álvarez‐Pérez, S. , K. Tsuji , M. Donald , et al. 2021. “Nitrogen Assimilation Varies Among Clades of Nectar‐ and Insect‐Associated Acinetobacters.” Microbial Ecology 81: 990–1003. 33404822 10.1007/s00248-020-01671-x

[ece371059-bib-0011] Barberis, M. , M. Nepi , and M. Galloni . 2024. “Floral Nectar: Fifty Years of New Ecological Perspectives Beyond Pollinator Reward.” Perspectives in Plant Ecology, Evolution and Systematics 62: 125764.

[ece371059-bib-0012] Bartlewicz, J. , M. I. Pozo , O. Honnay , B. Lievens , and H. Jacquemyn . 2016. “Effects of Agricultural Fungicides on Microorganisms Associated With Floral Nectar: Susceptibility Assays and Field Experiments.” Environmental Science and Pollution Research 23: 19776–19786. 27411538 10.1007/s11356-016-7181-4

[ece371059-bib-0013] Belisle, M. , K. G. Peay , and T. Fukami . 2012. “Flowers as Islands: Spatial Distribution of Nectar‐Inhabiting Microfungi Among Plants of *Mimulus aurantiacus*, a Hummingbird‐Pollinated Shrub.” Microbial Ecology 63: 711–718. 22080257 10.1007/s00248-011-9975-8 PMC4108428

[ece371059-bib-0014] Berg, M. P. , and J. Ellers . 2010. “Trait Plasticity in Species Interactions: A Driving Force of Community Dynamics.” Evolutionary Ecology 24: 617–629.

[ece371059-bib-0015] Bever, J. D. , I. A. Dickie , E. Facelli , et al. 2010. “Rooting Theories of Plant Community Ecology in Microbial Interactions.” Trends in Plant Science 25: 468–478. 10.1016/j.tree.2010.05.004 PMC2921684 20557974

[ece371059-bib-0016] Bochner, B. R. 2009. “Global Phenotypic Characterization of Bacteria.” FEMS Microbiology Reviews 33: 191–205. 19054113 10.1111/j.1574-6976.2008.00149.x PMC2704929

[ece371059-bib-0017] Borges, R. M. 2008. “Plasticity Comparisons Between Plants and Animals: Concepts and Mechanisms.” Plant Signaling & Behavior 3: 367–375. 19513224 10.4161/psb.3.6.5823 PMC2634305

[ece371059-bib-0018] Brysch‐Herzberg, M. 2004. “Ecology of Yeasts in Plant‐Bumblebee Mutualism in Central Europe.” FEMS Microbiology Ecology 50: 87–100. 19712367 10.1016/j.femsec.2004.06.003

[ece371059-bib-0019] Burton, T. , I. I. Ratikainen , and S. Einum . 2022. “Environmental Change and the Rate of Phenotypic Plasticity.” Global Change Biology 28: 5337–5345. 35729070 10.1111/gcb.16291 PMC9541213

[ece371059-bib-0020] Canto, A. , R. Pérez , M. Medrano , M. C. Castellanos , and C. M. Herrera . 2007. “Intra‐Plant Variation in Nectar Sugar Composition in Two *Aquilegia* Species (Ranunculaceae): Contrasting Patterns Under Field and Glasshouse Conditions.” Annals of Botany 99: 653–660. 17259227 10.1093/aob/mcl291 PMC2802931

[ece371059-bib-0021] Cecala, J. , L. Landucci , and R. L. Vannette . 2024. “Seasonal Assembly of Nectar Microbial Communities Across Angiosperm Plant Species: Assessing Contributions of Climate and Plant Traits.” Ecology Letters 28: e70045. 10.1111/ele.70045 PMC11687353 39737670

[ece371059-bib-0022] Celiker, H. , and J. Gore . 2012. “Competition Between Species Can Stabilize Public‐Goods Cooperation Within a Species.” Molecular Systems Biology 8: 621. 23149686 10.1038/msb.2012.54 PMC3531910

[ece371059-bib-0023] Chappell, C. R. , M. K. Dhami , M. C. Bitter , et al. 2022. “Wide‐Ranging Consequences of Priority Effects Governed by an Overarching Factor.” eLife 11: e79647. 36300797 10.7554/eLife.79647 PMC9671501

[ece371059-bib-0024] Chappell, C. R. , and T. Fukami . 2018. “Nectar Yeasts: A Natural Microcosm for Ecology.” Yeast 35: 417–423. 29476620 10.1002/yea.3311

[ece371059-bib-0025] Chappell, C. R. , P. C. Goddard , L. A. Golden , et al. 2024. “Transcriptional Responses to Priority Effects in Nectar Yeast.” Molecular Ecology 2024: e17553. 10.1111/mec.17553. 39450887

[ece371059-bib-0026] Chen, Z. , B. Mo , A. Lei , and J. Wang . 2022. “Microbial Single‐Cell Analysis: What Can We Learn From Mammalian?” Frontiers in Cell and Developmental Biology 9: 829990. 35111764 10.3389/fcell.2021.829990 PMC8801874

[ece371059-bib-0027] Crowley, B. , and A. Russell . 2021. “Plant Biology: Nectar Bacteria Grow by Germinating and Bursting Pollen.” Current Biology 31: R1120–R1122. 34637711 10.1016/j.cub.2021.08.024

[ece371059-bib-0028] Damore, J. A. , and J. Gore . 2012. “Understanding Microbial Cooperation.” Journal of Theoretical Biology 299: 31–41. 21419783 10.1016/j.jtbi.2011.03.008 PMC3176967

[ece371059-bib-0029] de Vega, C. , R. G. Albaladejo , S. Álvarez‐Pérez , and C. M. Herrera . 2022. “Contrasting Effects of Nectar Yeasts on the Reproduction of Mediterranean Plant Species.” American Journal of Botany 109: 393–405. 35315515 10.1002/ajb2.1834

[ece371059-bib-0030] de Vega, C. , S. Álvarez‐Pérez , R. G. Albaladejo , et al. 2021. “The Role of Plant–Pollinator Interactions in Structuring Nectar Microbial Communities.” Journal of Ecology 109: 3379–3395.

[ece371059-bib-0031] DeWitt, T. J. , and S. M. Scheiner . 2004. “Phenotypic Variation From Single Genotypes. A Primer.” In Phenotypic Plasticity: Functional and Conceptual Approaches, edited by T. J. DeWitt and S. M. Scheiner , 1–9. Oxford University Press.

[ece371059-bib-0032] DeWitt, T. J. , A. Sih , and D. S. Wilson . 1998. “Costs and Limits of Phenotypic Plasticity.” Trends in Ecology & Evolution 13: 77–81. 21238209 10.1016/s0169-5347(97)01274-3

[ece371059-bib-0033] Dhami, M. K. , T. Hartwig , and T. Fukami . 2016. “Genetic Basis of Priority Effects: Insights From Nectar Yeast.” Proceedings of the Royal Society B: Biological Sciences 283: 20161455. 10.1098/rspb.2016.1455 PMC5069511 27708148

[ece371059-bib-0034] Dhami, M. K. , T. Hartwig , A. D. Letten , M. Banf , and T. Fukami . 2018. “Genomic Diversity of a Nectar Yeast Clusters Into Metabolically, but Not Geographically, Distinct Lineages.” Molecular Ecology 27: 2067–2076. 29446179 10.1111/mec.14535

[ece371059-bib-0035] Donald, M. L. , J. A. Galbraith , D. A. Erastova , A. Podolyan , T. E. X. Miller , and M. K. Dhami . 2022. “Nectar Resources Affect Bird‐Dispersed Microbial Metacommunities in Suburban and Rural Gardens.” Environmental Microbiology 24: 5654–5665. 36102191 10.1111/1462-2920.16159 PMC10087401

[ece371059-bib-0036] D'Souza, G. G. 2020. “Phenotypic Variation in Spatially Structured Microbial Communities: Ecological Origins and Consequences.” Current Opinion in Biotechnology 62: 220–227. 31954366 10.1016/j.copbio.2019.12.013

[ece371059-bib-0037] Dupont, L. , M. Thierry , L. Zinger , D. Legrand , and S. Jacob . 2024. “Beyond Reaction Norms: The Temporal Dynamics of Phenotypic Plasticity.” Trends in Ecology & Evolution 39: 41–51. 37718228 10.1016/j.tree.2023.08.014

[ece371059-bib-0038] Duveau, F. , D. C. Yuan , B. P. H. Metzger , A. Hodgins‐Davis , and P. J. Wittkopp . 2017. “Effects of Mutation and Selection on Plasticity of a Promoter Activity in *Saccharomyces cerevisiae* .” Proceedings of the National Academy of Sciences of the United States of America 114: E11218–E11227. 29259117 10.1073/pnas.1713960115 PMC5748197

[ece371059-bib-0039] Escalas, A. , L. Hale , J. W. Voordeckers , et al. 2019. “Microbial Functional Diversity: From Concepts to Applications.” Ecology and Evolution 9: 12000–12016. 31695904 10.1002/ece3.5670 PMC6822047

[ece371059-bib-0040] Fink, J. W. , and M. Manhart . 2024. “Quantifying Microbial Fitness in High‐Throughput Experiments.” *bioRxiv*. 10.1101/2024.08.20.608874.

[ece371059-bib-0041] Forsman, A. 2015. “Rethinking Phenotypic Plasticity and Its Consequences for Individuals, Populations and Species.” Heredity 115: 276–284. 25293873 10.1038/hdy.2014.92 PMC4815454

[ece371059-bib-0042] Fox, R. J. , J. M. Donelson , C. Schunter , T. Ravasi , and J. D. Gaitán‐Espitia . 2019. “Beyond Buying Time: The Role of Plasticity in Phenotypic Adaptation to Rapid Environmental Change.” Philosophical Transactions of the Royal Society, B: Biological Sciences 374: 20180174. 30966962 10.1098/rstb.2018.0174 PMC6365870

[ece371059-bib-0043] Fraebel, D. T. , K. Gowda , M. Mani , and S. Kuehn . 2020. “Evolution of Generalists by Phenotypic Plasticity.” iScience 23: 101678. 33163936 10.1016/j.isci.2020.101678 PMC7600391

[ece371059-bib-0044] Friesen, M. L. , G. Saxer , M. Travisano , and M. Doebeli . 2004. “Experimental Evidence for Sympatric Ecological Diversification due to Frequency‐Dependent Competition in *Escherichia coli* .” Evolution 58: 245–260. 15068343

[ece371059-bib-0045] Fusco, G. , and A. Minelli . 2010. “Phenotypic Plasticity in Development and Evolution: Facts and Concepts.” Philosophical Transactions of the Royal Society, B: Biological Sciences 365: 547–556. 20083631 10.1098/rstb.2009.0267 PMC2817147

[ece371059-bib-0046] Gianoli, E. , and V. M. Escobedo . 2021. “Phenotypic Plasticity May Mediate Habitat Filtering in a Forest Edge Community.” Oikos 130: 1788–1796.

[ece371059-bib-0047] Goberna, M. , and M. Verdú . 2016. “Predicting Microbial Traits With Phylogenies.” ISME Journal 10: 959–967. 26371406 10.1038/ismej.2015.171 PMC4796935

[ece371059-bib-0048] Gómez, J. M. , A. González‐Megías , C. Armas , E. Narbona , L. Navarro , and F. Perfectti . 2023. “The Role of Phenotypic Plasticity in Shaping Ecological Networks.” Ecology Letters 26: S47–S61. 37840020 10.1111/ele.14192

[ece371059-bib-0049] Gomulkiewicz, R. , and J. R. Stinchcombe . 2022. “Phenotypic Plasticity Made Simple, but Not Too Simple.” American Journal of Botany 109: 1519–1524. 36109846 10.1002/ajb2.16068 PMC9828142

[ece371059-bib-0050] Good, A. P. , M. P. Gauthier , R. L. Vannette , and T. Fukami . 2014. “Honey Bees Avoid Nectar Colonized by Three Bacterial Species, but Not by a Yeast Species, Isolated From the Bee Gut.” PLoS One 9: e86494. 24466119 10.1371/journal.pone.0086494 PMC3899272

[ece371059-bib-0051] Harrington, K. I. , and A. Sanchez . 2014. “Eco‐Evolutionary Dynamics of Complex Social Strategies in Microbial Communities.” Communicative & Integrative Biology 7: e28230. 24778764 10.4161/cib.28230 PMC3995729

[ece371059-bib-0052] Hausmann, S. L. , B. Tietjen , and M. C. Rillig . 2017. “Solving the Puzzle of Yeast Survival in Ephemeral Nectar Systems: Exponential Growth Is Not Enough.” FEMS Microbiology Ecology 93: fix150. 10.1093/femsec/fix150 29106521

[ece371059-bib-0053] Hendry, A. P. 2016. “Key Questions on the Role of Phenotypic Plasticity in Eco‐Evolutionary Dynamics.” Journal of Heredity 107: 25–41. 26297912 10.1093/jhered/esv060

[ece371059-bib-0054] Herrera, C. M. 2014. “Population Growth of the Floricolous Yeast *Metschnikowia reukaufii*: Effects of Nectar Host, Yeast Genotype, and Host × Genotype Interaction.” FEMS Microbiology Ecology 88: 250–257. 24512559 10.1111/1574-6941.12284

[ece371059-bib-0055] Herrera, C. M. 2017. “Scavengers That Fit Beneath a Microscope Lens.” Ecology 98: 2725–2726. 28605015 10.1002/ecy.1874

[ece371059-bib-0056] Herrera, C. M. , A. Canto , M. I. Pozo , and P. Bazaga . 2010. “Inhospitable Sweetness: Nectar Filtering of Pollinator‐Borne Inocula Leads to Impoverished, Phylogenetically Clustered Yeast Communities.” Proceedings of the Royal Society B: Biological Sciences 277: 747–754. 10.1098/rspb.2009.1485 PMC2842741 19889702

[ece371059-bib-0057] Herrera, C. M. , I. M. García , and R. Pérez . 2008. “Invisible Floral Larcenies: Microbial Communities Degrade Floral Nectar of Bumblebee‐Pollinated Plants.” Ecology 89: 2369–2376. 18831156 10.1890/08-0241.1

[ece371059-bib-0058] Herrera, C. M. , R. Pérez , and C. Alonso . 2006. “Extreme Intraplant Variation in Nectar Sugar Composition in an Insect‐Pollinated Perennial Herb.” American Journal of Botany 93: 575–581. 21646218 10.3732/ajb.93.4.575

[ece371059-bib-0059] Herrera, C. M. , M. I. Pozo , and P. Bazaga . 2011. “Clonality, Genetic Diversity and Support for the Diversifying Selection Hypothesis in Natural Populations of a Flower‐Living Yeast.” Molecular Ecology 20: 4395–4407. 21851437 10.1111/j.1365-294X.2011.05217.x

[ece371059-bib-0060] Herrera, C. M. , M. I. Pozo , and P. Bazaga . 2012. “Jack of All Nectars, Master of Most: DNA Methylation and the Epigenetic Basis of Niche Width in a Flower‐Living Yeast.” Molecular Ecology 21: 2602–2616. 22171717 10.1111/j.1365-294X.2011.05402.x

[ece371059-bib-0061] Herrera, C. M. , M. I. Pozo , and P. Bazaga . 2014. “Nonrandom Genotype Distribution Among Floral Hosts Contributes to Local and Regional Genetic Diversity in the Nectar‐Living Yeast *Metschnikowia reukaufii* .” FEMS Microbiology Ecology 87: 568–575. 24283468 10.1111/1574-6941.12245

[ece371059-bib-0062] Herrera, C. M. , M. I. Pozo , and M. Medrano . 2013. “Yeasts in Nectar of an Early‐Blooming Herb: Sought by Bumble Bees, Detrimental to Plant Fecundity.” Ecology 94: 273–279. 23691645 10.1890/12-0595.1

[ece371059-bib-0063] Hu, T. , W. Banzhaf , and J. H. Moore . 2014. “The Effects of Recombination on Phenotypic Exploration and Robustness in Evolution.” Artificial Life 20: 457–470. 25148550 10.1162/ARTL_a_00145

[ece371059-bib-0064] Hurst, C. J. 2021. Microbes: The Foundation Stone of the Biosphere. Springer.

[ece371059-bib-0065] Jacquemyn, H. , M. I. Pozo , S. Álvarez‐Pérez , B. Lievens , and T. Fukami . 2021. “Yeast–Nectar Interactions: Metacommunities and Effects on Pollinators.” Current Opinion in Insect Science 44: 35–40. 33065340 10.1016/j.cois.2020.09.014

[ece371059-bib-0066] Kamilar, J. M. , and N. Cooper . 2013. “Phylogenetic Signal in Primate Behaviour, Ecology and Life History.” Philosophical Transactions of the Royal Society, B: Biological Sciences 368: 20120341. 23569289 10.1098/rstb.2012.0341 PMC3638444

[ece371059-bib-0067] Keeling, P. J. 2024. “Horizontal Gene Transfer in Eukaryotes: Aligning Theory With Data.” Nature Reviews Genetics 25: 416–430. 10.1038/s41576-023-00688-5 38263430

[ece371059-bib-0068] Klaps, J. , B. Lievens , and S. Álvarez‐Pérez . 2020. “Towards a Better Understanding of the Role of Nectar‐Inhabiting Yeasts in Plant–Animal Interactions.” Fungal Biology and Biotechnology 7: 1. 31921433 10.1186/s40694-019-0091-8 PMC6947986

[ece371059-bib-0069] Kost, C. , K. R. Patil , J. Friedman , S. L. Garcia , and M. Ralser . 2023. “Metabolic Exchanges Are Ubiquitous in Natural Microbial Communities.” Nature Microbiology 8: 2244–2252. 10.1038/s41564-023-01511-x 37996708

[ece371059-bib-0070] Kovuri, P. , A. Yadav , and H. Sinha . 2023. “Role of Genetic Architecture in Phenotypic Plasticity.” Trends in Genetics 39: 703–714. 37173192 10.1016/j.tig.2023.04.002

[ece371059-bib-0071] Krause, S. , X. Le Roux , P. A. Niklaus , et al. 2014. “Trait‐Based Approaches for Understanding Microbial Biodiversity and Ecosystem Functioning.” Frontiers in Microbiology 5: 251. 24904563 10.3389/fmicb.2014.00251 PMC4033906

[ece371059-bib-0072] Kümmerli, R. , N. Jiricny , L. S. Clarke , S. A. West , and A. S. Griffin . 2009. “Phenotypic Plasticity of a Cooperative Behaviour in Bacteria.” Journal of Evolutionary Biology 22: 589–598. 19170825 10.1111/j.1420-9101.2008.01666.x

[ece371059-bib-0073] Lachance, M. A. 2016. “ *Metschnikowia*: Half Tetrads, a Regicide and the Fountain of Youth.” Yeast 33: 563–574. 27599462 10.1002/yea.3208

[ece371059-bib-0074] Landucci, L. , and R. L. Vannette . 2024. “Plant Species Vary in Nectar Peroxide, an Antimicrobial Defense Tolerated by Some Bacteria and Yeasts.” *bioRxiv*. 10.1101/2024.10.25.620320.

[ece371059-bib-0076] Laviad‐Shitrit, S. , I. Izhaki , W. B. Whitman , et al. 2020. “Draft Genome of *Rosenbergiella nectarea* Strain 8N4^T^ Provides Insights Into the Potential Role of This Species in Its Plant Host.” PeerJ 8: e8822. 32292647 10.7717/peerj.8822 PMC7144588

[ece371059-bib-0077] Lenaerts, M. , S. Álvarez‐Pérez , C. de Vega , et al. 2014. “ *Rosenbergiella australoborealis* sp. Nov., *Rosenbergiella collisarenosi* sp. Nov. and *Rosenbergiella epipactidis* sp. Nov., Three Novel Bacterial Species Isolated From Floral Nectar.” Systematic and Applied Microbiology 37: 402–411. 24794950 10.1016/j.syapm.2014.03.002

[ece371059-bib-0078] Lenaerts, M. , T. Goelen , C. Paulussen , et al. 2017. “Nectar Bacteria Affect Life History of a Generalist Aphid Parasitoid by Altering Nectar Chemistry.” Functional Ecology 31: 2061–2069.

[ece371059-bib-0079] Lievens, B. , J. E. Hallsworth , M. I. Pozo , et al. 2015. “Microbiology of Sugar‐Rich Environments: Diversity, Ecology and System Constraints.” Environmental Microbiology 17: 278–298. 25041632 10.1111/1462-2920.12570

[ece371059-bib-0080] Liu, Y. , S. Dunker , W. Durka , et al. 2024. “Eco‐Evolutionary Processes Shaping Floral Nectar Sugar Composition.” Scientific Reports 14: 13856. 38879632 10.1038/s41598-024-64755-5 PMC11180116

[ece371059-bib-0081] Mallard, F. , V. Nolte , and C. Schlötterer . 2020. “The Evolution of Phenotypic Plasticity in Response to Temperature Stress.” Genome Biology and Evolution 12: 2429–2440. 33022043 10.1093/gbe/evaa206 PMC7846148

[ece371059-bib-0082] Martiny, J. B. , S. E. Jones , J. T. Lennon , and A. C. Martiny . 2015. “Microbiomes in Light of Traits: A Phylogenetic Perspective.” Science 350: aac9323. 26542581 10.1126/science.aac9323

[ece371059-bib-0083] Matesanz, S. , M. Blanco‐Sánchez , M. Ramos‐Muñoz , M. de la Cruz , R. Benavides , and A. Escudero . 2021. “Phenotypic Integration Does Not Constrain Phenotypic Plasticity: Differential Plasticity of Traits Is Associated to Their Integration Across Environments.” New Phytologist 231: 2359–2370. 34097309 10.1111/nph.17536

[ece371059-bib-0084] Meroz, N. , T. Livny , and J. Friedman . 2024. “Quantifying Microbial Interactions: Concepts, Caveats, and Applications.” Current Opinion in Microbiology 80: 102511. 39002491 10.1016/j.mib.2024.102511

[ece371059-bib-0085] Morales‐Poole, J. R. , C. de Vega , K. Tsuji , et al. 2023. “Sugar Concentration, Nitrogen Availability, and Phylogenetic Factors Determine the Ability of *Acinetobacter* spp. and *Rosenbergiella* spp. to Grow in Floral Nectar.” Microbial Ecology 86: 377–391. 35930073 10.1007/s00248-022-02088-4 PMC10293439

[ece371059-bib-0086] Morris, M. M. , N. J. Frixione , A. C. Burkert , E. A. Dinsdale , and R. L. Vannette . 2020. “Microbial Abundance, Composition, and Function in Nectar Are Shaped by Flower Visitor Identity.” FEMS Microbiology Ecology 96: fiaa003. 31922546 10.1093/femsec/fiaa003

[ece371059-bib-0087] Moxon, R. , and E. Kussell . 2017. “The Impact of Bottlenecks on Microbial Survival, Adaptation, and Phenotypic Switching in Host‐Pathogen Interactions.” Evolution 71: 2803–2816. 28983912 10.1111/evo.13370 PMC5722657

[ece371059-bib-0088] Mueller, T. G. , J. S. Francis , and R. L. Vannette . 2023. “Nectar Compounds Impact Bacterial and Fungal Growth and Shift Community Dynamics in a Nectar Analog.” Environmental Microbiology Reports 15: 170–180. 36779256 10.1111/1758-2229.13139 PMC10464699

[ece371059-bib-0089] Nemergut, D. R. , S. K. Schmidt , T. Fukami , et al. 2013. “Patterns and Processes of Microbial Community Assembly.” Microbiology and Molecular Biology Reviews 77: 342–356. 24006468 10.1128/MMBR.00051-12 PMC3811611

[ece371059-bib-0090] Nguyen, V. , Y. Li , and T. Lu . 2024. “Emergence of Orchestrated and Dynamic Metabolism of *Saccharomyces cerevisiae* .” ACS Synthetic Biology 13: 1442–1453. 38657170 10.1021/acssynbio.3c00542 PMC11103795

[ece371059-bib-0091] Nicolson, S. W. 2022. “Sweet Solutions: Nectar Chemistry and Quality.” Philosophical Transactions of the Royal Society, B: Biological Sciences 377: 20210163. 35491604 10.1098/rstb.2021.0163 PMC9058545

[ece371059-bib-0092] Nicolson, S. W. , and R. W. Thornburg . 2007. “Nectar Chemistry.” In Nectaries and Nectar, edited by S. W. Nicolson , M. Nepi , and E. Pacini , 215–264. Springer‐Verlag.

[ece371059-bib-0093] Nielsen, M. E. , and D. R. Papaj . 2022. “Why Study Plasticity in Multiple Traits? New Hypotheses for How Phenotypically Plastic Traits Interact During Development and Selection.” Evolution 76: 858–869. 35274745 10.1111/evo.14464 PMC9313899

[ece371059-bib-0094] Niu, H. , J. Gu , and Y. Zhang . 2024. “Bacterial Persisters: Molecular Mechanisms and Therapeutic Development.” Signal Transduction and Targeted Therapy 9: 174. 39013893 10.1038/s41392-024-01866-5 PMC11252167

[ece371059-bib-0095] Oliveira, P. H. 2021. “Bacterial Epigenomics: Coming of Age.” mSystems 6: e0074721. 34402642 10.1128/mSystems.00747-21 PMC8407109

[ece371059-bib-0096] Ørsted, M. , P. D. Rohde , A. A. Hoffmann , P. Sørensen , and T. N. Kristensen . 2018. “Environmental Variation Partitioned Into Separate Heritable Components.” Evolution 72: 136–152. 29125643 10.1111/evo.13391

[ece371059-bib-0097] Palacio‐López, K. , B. Beckage , S. Scheiner , and J. Molofsky . 2015. “The Ubiquity of Phenotypic Plasticity in Plants: A Synthesis.” Ecology and Evolution 5: 3389–3400. 26380672 10.1002/ece3.1603 PMC4569034

[ece371059-bib-0098] Palmer‐Young, E. C. , I. W. Farrell , L. S. Adler , et al. 2019. “Chemistry of Floral Rewards: Intra‐ and Interspecific Variability of Nectar and Pollen Secondary Metabolites Across Taxa.” Ecological Monographs 89: e01335.

[ece371059-bib-0099] Parachnowitsch, A. L. , J. S. Manson , and N. Sletvold . 2019. “Evolutionary Ecology of Nectar.” Annals of Botany 123: 247–261. 30032269 10.1093/aob/mcy132 PMC6344224

[ece371059-bib-0100] Peay, K. G. , M. Belisle , and T. Fukami . 2012. “Phylogenetic Relatedness Predicts Priority Effects in Nectar Yeast Communities.” Proceedings of the Royal Society B: Biological Sciences 279, no. 1729: 749–758. 10.1098/rspb.2011.1230 PMC3248732 21775330

[ece371059-bib-0101] Peltier, E. , V. Sharma , M. Martí Raga , et al. 2018. “Dissection of the Molecular Bases of Genotype × Environment Interactions: A Study of Phenotypic Plasticity of *Saccharomyces cerevisiae* in Grape Juices.” BMC Genomics 19: 772. 30409183 10.1186/s12864-018-5145-4 PMC6225642

[ece371059-bib-0102] Pérez‐Ramos, I. M. , L. Matías , L. Gómez‐Aparicio , and Ó. Godoy . 2019. “Functional Traits and Phenotypic Plasticity Modulate Species Coexistence Across Contrasting Climatic Conditions.” Nature Communications 10: 2555. 10.1038/s41467-019-10453-0 PMC6560116 31186418

[ece371059-bib-0103] Pope, C. F. , T. D. McHugh , and S. H. Gillespie . 2010. “Methods to Determine Fitness in Bacteria.” Methods in Molecular Biology 642: 113–121. 20401590 10.1007/978-1-60327-279-7_9

[ece371059-bib-0104] Pozo, M. I. , C. M. Herrera , and P. Bazaga . 2011. “Species Richness of Yeast Communities in Floral Nectar of Southern Spanish Plants.” Microbial Ecology 61: 82–91. 20449581 10.1007/s00248-010-9682-x

[ece371059-bib-0105] Pozo, M. I. , C. M. Herrera , M. A. Lachance , K. Verstrepen , B. Lievens , and H. Jacquemyn . 2016. “Species Coexistence in Simple Microbial Communities: Unravelling the Phenotypic Landscape of Co‐Occurring *Metschnikowia* Species in Floral Nectar.” Environmental Microbiology 18: 1850–1862. 26337395 10.1111/1462-2920.13037

[ece371059-bib-0106] Pozo, M. I. , C. M. Herrera , W. van den Ende , K. Verstrepen , B. Lievens , and H. Jacquemyn . 2015. “The Impact of Nectar Chemical Features on Phenotypic Variation in Two Related Nectar Yeasts.” FEMS Microbiology Ecology 91: fiv055. 25994159 10.1093/femsec/fiv055

[ece371059-bib-0107] Pozo, M. I. , and H. Jacquemyn . 2019. “Addition of Pollen Increases Growth of Nectar‐Living Yeasts.” FEMS Microbiology Letters 366: fnz191. 31550375 10.1093/femsle/fnz191

[ece371059-bib-0108] Pozo, M. I. , M. A. Lachance , and C. M. Herrera . 2012. “Nectar Yeasts of Two Southern Spanish Plants: The Roles of Immigration and Physiological Traits in Community Assembly.” FEMS Microbiology Ecology 80: 281–293. 22224447 10.1111/j.1574-6941.2011.01286.x

[ece371059-bib-0109] Pozo, M. I. , B. Lievens , and H. Jacquemyn . 2015. “Impact of Microorganisms on Nectar Chemistry, Pollinator Attraction and Plant Fitness.” In Nectar: Production, Chemical Composition and Benefits to Animals and Plants, edited by R. L. Peck , 1–40. Nova Science Publishers Inc.

[ece371059-bib-0110] Prosser, J. I. , B. J. M. Bohannan , T. P. Curtis , et al. 2007. “The Role of Ecological Theory in Microbial Ecology.” Nature Reviews Microbiology 5: 384–392. 17435792 10.1038/nrmicro1643

[ece371059-bib-0111] Quevedo‐Caraballo, S. , C. de Vega , B. Lievens , T. Fukami , and S. Álvarez‐Pérez . 2025. “Tiny but Mighty? Overview of a Decade of Research on Nectar Bacteria.” New Phytologist 245, no. 5: 1897–1910. 10.1111/nph.20369. 39716780 PMC11798911

[ece371059-bib-0112] Quevedo‐Caraballo, S. , A. Roldán , and S. Álvarez‐Pérez . 2024. “Demethylation Inhibitor Fungicides Have a Significantly Detrimental Impact on Population Growth and Composition of Nectar Microbial Communities.” Microbial Ecology 87: 160. 39708144 10.1007/s00248-024-02477-x PMC11663151

[ece371059-bib-0113] Reed, T. E. , R. S. Waples , D. E. Schindler , J. J. Hard , and M. T. Kinnison . 2010. “Phenotypic Plasticity and Population Viability: The Importance of Environmental Predictability.” Proceedings of the Royal Society B: Biological Sciences 277: 3391–3400. 10.1098/rspb.2010.0771 PMC2982227 20554553

[ece371059-bib-0114] Rering, C. C. , J. J. Beck , G. W. Hall , M. M. McCartney , and R. L. Vannette . 2018. “Nectar‐Inhabiting Microorganisms Influence Nectar Volatile Composition and Attractiveness to a Generalist Pollinator.” New Phytologist 220: 750–759. 28960308 10.1111/nph.14809

[ece371059-bib-0115] Rering, C. C. , R. L. Vannette , R. N. Schaeffer , and J. J. Beck . 2020. “Microbial Co‐Occurrence in Floral Nectar Affects Metabolites and Attractiveness to a Generalist Pollinator.” Journal of Chemical Ecology 46: 659–667. 32246258 10.1007/s10886-020-01169-3

[ece371059-bib-0116] San Roman, M. , and A. Wagner . 2018. “An Enormous Potential for Niche Construction Through Bacterial Cross‐Feeding in a Homogeneous Environment.” PLoS Computational Biology 14: e1006340. 30040834 10.1371/journal.pcbi.1006340 PMC6080805

[ece371059-bib-0117] Sanchez, V. A. , T. Renner , L. J. Baker , and T. A. Hendry . 2025. “Genome Evolution Following an Ecological Shift in Nectar‐Dwelling.” mSphere 10, no. 1: e01010‐24. 10.1128/msphere.01010-24. 39723821 PMC11774029

[ece371059-bib-0118] Sánchez‐Romero, M. A. , and J. Casadesús . 2020. “The Bacterial Epigenome.” Nature Reviews Microbiology 18: 7–20. 31728064 10.1038/s41579-019-0286-2

[ece371059-bib-0119] Santiago, E. , D. F. Moreno , and M. Acar . 2022. “Phenotypic Plasticity as a Facilitator of Microbial Evolution.” Environmental Epigenetics 8: dvac020. 36465837 10.1093/eep/dvac020 PMC9709823

[ece371059-bib-0120] Schaeffer, R. N. , and R. E. Irwin . 2014. “Yeasts in Nectar Enhance Male Fitness in a Montane Perennial Herb.” Ecology 95: 1792–1798. 25163113 10.1890/13-1740.1

[ece371059-bib-0121] Schaeffer, R. N. , C. C. Rering , I. Maalouf , J. J. Beck , and R. L. Vannette . 2019. “Microbial Metabolites Elicit Distinct Olfactory and Gustatory Preferences in Bumblebees.” Biology Letters 15: 20190132. 31311487 10.1098/rsbl.2019.0132 PMC6684982

[ece371059-bib-0122] Schrey, A. W. , and C. L. Richards . 2012. “Within‐Genotype Epigenetic Variation Enables Broad Niche Width in a Flower Living Yeast.” Molecular Ecology 21: 2559–2561. 22624945 10.1111/j.1365-294X.2012.05487.x

[ece371059-bib-0123] Scott, M. , and T. Hwa . 2023. “Shaping Bacterial Gene Expression by Physiological and Proteome Allocation Constraints.” Nature Reviews Microbiology 21: 327–342. 36376406 10.1038/s41579-022-00818-6 PMC10121745

[ece371059-bib-0124] Sharaby, Y. , S. Rodríguez‐Martínez , M. Lalzar , M. Halpern , and I. Izhaki . 2020. “Geographic Partitioning or Environmental Selection: What Governs the Global Distribution of Bacterial Communities Inhabiting Floral Nectar?” Science of the Total Environment 749: 142305. 33370885 10.1016/j.scitotenv.2020.142305

[ece371059-bib-0125] Sipiczki, M. , and K. Czentye . 2024. “Reversible Stochastic Epigenetic Like Silencing of the Production of Pulcherriminic Acid in the Antimicrobial Antagonist *Metschnikowia pulcherrima* .” Scientific Reports 14: 29677. 39613864 10.1038/s41598-024-80436-9 PMC11607323

[ece371059-bib-0126] Sipiczki, M. , K. Czentye , and Z. Kállai . 2024. “High Intragenomic, Intergenomic, and Phenotypic Diversity in Pulcherrimin‐Producing *Metschnikowia* Yeasts Indicates a Special Mode of Genome Evolution.” Scientific Reports 14: 10521. 38714828 10.1038/s41598-024-61335-5 PMC11076541

[ece371059-bib-0127] Stroud, J. T. , B. M. Delory , E. M. Barnes , et al. 2024. “Priority Effects Transcend Scales and Disciplines in Biology.” Trends in Ecology & Evolution 39: 677–688. 38508922 10.1016/j.tree.2024.02.004

[ece371059-bib-0128] Suzuki, R. , and T. Arita . 2014. “Emergence of a Dynamic Resource Partitioning Based on the Coevolution of Phenotypic Plasticity in Sympatric Species.” Journal of Theoretical Biology 352: 51–59. 24607740 10.1016/j.jtbi.2014.02.035

[ece371059-bib-0129] Tucker, C. M. , and T. Fukami . 2014. “Environmental Variability Counteracts Priority Effects to Facilitate Species Coexistence: Evidence From Nectar Microbes.” Proceedings of the Royal Society B: Biological Sciences 281: 20132637. 10.1098/rspb.2013.2637 PMC3906935 24430846

[ece371059-bib-0130] van Boxtel, C. , J. H. van Heerden , N. Nordholt , P. Schmidt , and F. J. Bruggeman . 2017. “Taking Chances and Making Mistakes: Non‐Genetic Phenotypic Heterogeneity and Its Consequences for Surviving in Dynamic Environments.” Journal of the Royal Society, Interface 14: 20170141. 28701503 10.1098/rsif.2017.0141 PMC5550968

[ece371059-bib-0131] Van Buskirk, J. , and U. K. Steiner . 2009. “The Fitness Costs of Developmental Canalization and Plasticity.” Journal of Evolutionary Biology 22: 852–860. 19226418 10.1111/j.1420-9101.2009.01685.x

[ece371059-bib-0132] Vannette, R. L. 2020. “The Floral Microbiome: Plant, Pollinator, and Microbial Perspectives.” Annual Review of Ecology, Evolution, and Systematics 51: 363–386.

[ece371059-bib-0133] Vannette, R. L. , and T. Fukami . 2014. “Historical Contingency in Species Interactions: Towards Niche‐Based Predictions.” Ecology Letters 17: 115–124. 24341984 10.1111/ele.12204 PMC4344821

[ece371059-bib-0134] Vannette, R. L. , and T. Fukami . 2016. “Nectar Microbes Can Reduce Secondary Metabolites in Nectar and Alter Effects on Nectar Consumption by Pollinators.” Ecology 97: 1410–1419. 27459772 10.1890/15-0858.1

[ece371059-bib-0135] Vannette, R. L. , and T. Fukami . 2017. “Dispersal Enhances Beta Diversity in Nectar Microbes.” Ecology Letters 20: 901–910. 28597955 10.1111/ele.12787

[ece371059-bib-0136] Vannette, R. L. , and T. Fukami . 2018. “Contrasting Effects of Yeasts and Bacteria on Floral Nectar Traits.” Annals of Botany 121: 1343–1349. 29562323 10.1093/aob/mcy032 PMC6007235

[ece371059-bib-0137] Vannette, R. L. , M. P. Gauthier , and T. Fukami . 2013. “Nectar Bacteria, but Not Yeast, Weaken a Plant‐Pollinator Mutualism.” Proceedings of the Royal Society B: Biological Sciences 280: 20122601. 10.1098/rspb.2012.2601 PMC3574316 23222453

[ece371059-bib-0138] Vannette, R. L. , M. S. McMunn , G. W. Hall , T. G. Mueller , I. Munkres , and D. Perry . 2021. “Culturable Bacteria Are More Common Than fungi in Floral Nectar and Are More Easily Dispersed by thrips, a Ubiquitous Flower Visitor.” FEMS Microbiology Ecology 97: fiab150. 34791198 10.1093/femsec/fiab150

[ece371059-bib-0139] Vogt, G. 2023. “Environmental Adaptation of Genetically Uniform Organisms With the Help of Epigenetic Mechanisms‐An Insightful Perspective on Ecoepigenetics.” Epigenomes 7, no. 1: 1. 10.3390/epigenomes7010001 PMC9844400 36648862

[ece371059-bib-0140] Wagner, A. 2011. “The Low Cost of Recombination in Creating Novel Phenotypes: Recombination Can Create New Phenotypes While Disrupting Well‐Adapted Phenotypes Much Less Than Mutation.” BioEssays 33: 636–646. 21633964 10.1002/bies.201100027

[ece371059-bib-0141] Wein, T. , and T. Dagan . 2019. “The Effect of Population Bottleneck Size and Selective Regime on Genetic Diversity and Evolvability in Bacteria.” Genome Biology and Evolution 11: 3283–3290. 31688900 10.1093/gbe/evz243 PMC7145630

[ece371059-bib-0142] Yang, M. , G. C. Deng , Y. B. Gong , and S. Q. Huang . 2019. “Nectar Yeasts Enhance the Interaction Between *Clematis akebioides* and Its Bumblebee Pollinator.” Plant Biology 21: 732–737. 30636362 10.1111/plb.12957

[ece371059-bib-0143] Zhu, M. , and X. Dai . 2024. “Shaping of Microbial Phenotypes by Trade‐Offs.” Nature Communications 15: 4238. 10.1038/s41467-024-48591-9 PMC11102524 38762599

